# Different effects of medications for hypertension on renal function between patients with and without diabetes mellitus undergoing percutaneous coronary intervention: a retrospective single-center cohort study

**DOI:** 10.1186/s12872-023-03547-7

**Published:** 2023-10-14

**Authors:** Daisuke Kanda, Takuro Takumi, Akihiro Tokushige, Yoshiyuki Ikeda, Mitsuru Ohishi

**Affiliations:** Department of Cardiovascular Medicine and Hypertension, Graduate School of Medical and Dental Sciences, Kagoshima University, 8-35-1 Sakuragaoka, Kagoshima City, Kagoshima, 890-8520 Japan

**Keywords:** β-blocker, Diabetes mellitus, Hypertension, Cystatin C, Renal function

## Abstract

**Background:**

Diabetes mellitus (DM) and hypertension are well-known atherosclerosis risk factors. Furthermore, renal dysfunction is a crucial risk factor for patients with coronary artery disease (CAD), and managing renal function in these patients is complicated because of comorbid conditions and potential side effects during treatment. Therefore, this study aimed to investigate the effect of medications for hypertension on renal function after percutaneous coronary intervention (PCI) between patients with and without DM with statins.

**Methods:**

In 297 consecutive patients undergoing PCI for stable angina pectoris, cystatin C (CysC) was evaluated at baseline and 9 months after PCI, and the percent change in CysC (%CysC) was calculated. The association of worsening renal function (WRF: %CysC ≥ 0) and baseline characteristics, including medications, was assessed.

**Results:**

Among 297 hypertensive patients with statins, 196 and 101 were with and without DM, respectively. Angiotensin-converting enzyme inhibitor (ACEI), angiotensin II receptor blocker, and β-blocker were prescribed in 56 (29%), 82 (42%), and 91 (46%) patients in the DM group, and 20 (20%), 52 (51%), and 52 (51%) in the non-DM group, respectively. The patients with WRF after PCI were 100 (51%) and 59 (58%) in the DM and non-DM groups (*p* = 0.261). Additionally, the %CysC had no significant differences between groups [median: 0%, interquartile range (IQR): -7.9% to 8.5% vs. median: 1.1%, IQR: -6.6% to 9.6%, *p* = 0.521]. Multivariate logistic analysis for WRF using relevant factors from univariate analysis showed that only β-blocker [odds ratio (OR): 2.76, 95% confidence interval (CI): 1.03–7.90, *p* = 0.048] was independently associated with WRF in the DM group whereas ACEI (OR: 0.07, 95% CI: 0.01–0.47, *p* = 0.012) was negatively correlated with WRF in the non-DM group.

**Conclusion:**

The β-blocker was the independent risk factor for WRF in patients with DM in the late phase after PCI for stable angina pectoris, while the use of ACEI had a renoprotective effect in patients without DM.

## Background

Recently, percutaneous coronary intervention (PCI) has become a pivotal treatment for patients with coronary artery disease (CAD), and complete revascularization via PCI has reportedly resulted in lower long-term mortality [1] even in patients with diabetes mellitus (DM) and multivessel coronary disease. However, acute kidney injury (AKI) after PCI is associated with an increase in mortality [2, 3], and Murata et al. also reported that persistent worsening renal function (WRF) is associated with a high incidence of all-cause mortality and major adverse cardiac events in patients who underwent PCI for acute coronary syndrome [4]. Moreover, chronic kidney disease (CKD) is an independent risk factor for cardiovascular events and all-cause mortality, predicting cardiovascular events as effectively as other established risk factors [5, 6].

We reported that angiotensin-converting enzyme inhibitors (ACEI) might have a preventive effect on WRF assessed by serum cystatin C (CysC) measurements in the late phase after PCI [7], and ACEI, angiotensin II receptor blockers (ARB), and β-blockers have been shown to lower cardiovascular mortality in the populations with cardiovascular disease. Statin therapy is also commonly used for the secondary prevention of cardiovascular disease. A previous study reported that statin therapy appears to reduce proteinuria and CKD progression rate [8]*.* Additionally, a meta-analysis of patients undergoing coronary angiography or PCI showed that short-term statin therapy reduced contrast-induced nephropathy (CIN) incidence and that the use of statins should be considered even in patients with reduced low-density lipoprotein cholesterol (LDL-C) levels [9]. However, previous studies reported that DM and hypertension were known as strong risk factors for the incidence of CIN.

Therefore, this study aimed to investigate the differences in the effects of medications on renal function in the late phase after PCI between hypertensive patients with and without DM among those with statins. Furthermore, we included additional patients following our previous report [7] and investigated the change in renal function before and 9 months after PCI by measuring serum CysC and factors affecting renal function.

## Methods

### Study population

This retrospective single-center cohort study included 297 consecutive patients with hypertension who underwent elective PCI for stable angina pectoris at Kagoshima University Hospital from January 2010 to July 2016. All patients received statin therapy regardless of dyslipidemia before the PCI procedure and underwent follow-up coronary angiography 9 months after PCI, excluding those who could not be tracked after discharge. Patients with cardiogenic shock or those treated with intra-aortic balloon pumping during PCI were excluded from this study since they are risk factors for AKI. Furthermore, patients undergoing hemodialysis were also excluded. This study was approved by the Research and Ethics Committee at Kagoshima University Hospital and was conducted in accordance with the ethical principles stated in the 1975 Declaration of Helsinki. All patients provided written informed consent before enrolment.

### PCI procedure

All patients received dual antiplatelet therapy and intravenous heparin before the procedure. In patients with serum creatinine (Cr) levels > 1.2 mg/dL, intravenous hydration with normal saline was provided at 1 mL/kg/h for 12 h before and after PCI. A nonionic iso-osmolar contrast media was used during PCI. Finally, all patients underwent a follow-up coronary angiography 9 months after PCI.

### Assessment of renal function

Laboratory values were obtained on admission before PCI. Serum CysC was measured using a colloidal gold particle-enhanced colorimetric immunoassay (Nescauto GC Cystatin C, Alfresa Pharma, Osaka, Japan). The estimated glomerular filtration rate (eGFR) was calculated using the Modification of Diet in Renal Disease equation, with coefficients modified for the Japanese population as follows: eGFR (mL/min/1.73 m^2^) = 194 × serum Cr (mg/dL)^−1.094^ × age (years)^−0.287^ (× 0.739 for female participants) [10]. The grade of renal function was classified based on the level of eGFR [11]. Serum concentrations of CysC were reassessed 9 months after PCI. We evaluated renal function change 9 months after PCI, which was defined as the late phase when patients received the follow-up coronary angiography. The percent change in CysC [%CysC = (CysC at 9 months—CysC at baseline)/CysC at baseline × 100] was calculated. Furthermore, %CysC ≥ 0% and %CysC < 0% after PCI were defined as WRF and non-WRF, respectively.

### Definitions

DM was defined based on the following criteria: use of antihyperglycemic medication, fasting plasma glucose concentration > 126 mg/dL, or glycated hemoglobin concentration ≥ 6.5% (in accordance with the National Glycohemoglobin Standardization Program) [12].

### Statistical analysis

Quantitative data are presented as mean ± standard deviation or median and interquartile range (IQR). Fisher’s exact test was used to compare the incidence of categorical variables, which are expressed as percentages. Continuous variables were compared between the DM and non-DM groups using Student’s t-test or the Wilcoxon rank-sum test for normal or non-normal distribution data, respectively. Univariate logistic regression analysis was performed using medication details known at the time of admission, patient background, and common comorbidities associated with cardiovascular disease as parameters. We performed multivariate logistic analysis for the presence of WRF using relevant factors from univariate analysis, with the results expressed as the odds ratio (OR) and 95% confidence interval (CI). Statistical significance was considered at *p* < 0.05, and statistical analyses were performed using SAS software (JMP® 16 (SAS Institute Inc., Cary, NC, USA).

## Results

### Baseline characteristics

Table 1 shows the baseline clinical characteristics of patients. Among 297 hypertensive patients with statins, 196 and 101 were with and without DM, respectively. Significant differences in diastolic blood pressure (median: 73 mmHg, IQR: 69–81 mmHg vs. median: 70 mmHg, IQR: 64–77 mmHg, *p* = 0.003), hemoglobin (median: 13.0 g/dL, IQR: 11.8–14.1 g/dL vs. median: 13.2 g/dL, IQR: 12.1–14.7 mg/dL, *p* = 0.030), high-density lipoprotein cholesterol (HDL-C) (median: 47 mg/dL, IQR: 38–54 mg/dL vs. median: 49 mg/dL, IQR 41–61 mg/dL, *p* = 0.025), fasting plasma glucose (median: 114 mg/dL, IQR: 99–148 mg/dL vs. median: 93 mg/dL, IQR: 89–106 mg/dL), *p* < 0.001, and glycated hemoglobin (median: 6.8%, IQR: 6.1–7.3% vs. median: 5.9%, IQR: 5.6–6.1%, *p* < 0.001) levels were found between the DM and non-DM groups.

**Table 1:** Patient characteristics

	Overall *n* = 297	DM, n (%) = 196 (66)	non-DM, n (%) = 101 (34)	*P*-value (DM vs. non-DM)
Age, y	69 [62, 75]	69 [63, 75]	70 [58, 76]	0.843
Body mass index, kg/m^2^	24.1 [22.2, 26.3]	24.8 [22.4, 26.3]	23.8 [22.1, 26.3]	0.245
Gender: men, n (%)	215 (72)	142 (72)	73 (72)	1.000
Contrast media, mL	150 [124, 182]	150 [125, 180]	150 [121, 189]	0.544
Systolic blood pressure, mmHg	128 [117, 138]	128 [116, 140]	128 [118, 138]	0.977
Diastolic blood pressure, mmHg	71 [65, 79]	73 [69, 81]	70 [64, 77]	0.003
Risk factors, n (%)				
DM	196 (66)	196 (100)	0 (0)	-
Dyslipidemia	225 (76)	147 (65)	78 (35)	0.671
Hyperuricemia	78 (26)	45 (23)	33 (33)	0.095
Medication, n (%)				
Calcium channel blockers	163 (55)	112 (57)	51 (50)	0.269
ACEI	76 (26)	56 (29)	20 (20)	0.123
ARB	134 (45)	82 (42)	52 (51)	0.176
β-blockers	143 (48)	91 (46)	52 (51)	0.394
Spironolactone	35 (12)	24 (12)	11 (11)	0.850
Thiazide	12 (4)	8 (4)	4 (4)	1.000
Loop diuretics	56 (19)	41 (21)	15 (15)	0.273
Diuretics	74 (25)	53 (27)	21 (21)	0.261
eGFR, mL/min/1.73 m^2^	63 [47,76]	64 [45,76]	62 [48,74]	0.676
G1 (> 90)	21 (7)	13 (7)	8 (8)	
G2 (60–89)	140 (47)	96 (49)	44 (44)	
G3a (45–59)	75 (25)	41 (21)	34 (34)	
G3b (30–44)	51 (17)	39 (20)	12 (12)	
G4 (15–29)	9 (3)	7 (3)	2 (2)	
G5 (< 15)	1 (0.3)	0 (0)	1 (1)	
Hb, g/dL	13.1 [11.9, 14.3]	13.0 [11.8, 14.1]	13.2 [12.1, 14.7]	0.030
hs-CRP, mg /dL	0.09 [0.04, 0.22]	0.08 [0.04, 0.21]	0.10 [0.05, 0.25]	0.387
LDL-C, mg/dL	84 [68, 101]	83 [67, 103]	85 [70, 100]	0.706
HDL-C, mg/dL	47 [39, 57]	47 [38, 54]	49 [41, 61]	0.025
TG, mg/dL	115 [84, 164]	115 [87, 164]	109 [81, 164]	0.567
UA, mg/dL	6.0 [5.0, 7.0]	5.9 [5.0, 6.8]	6.0 [5.0, 7.3]	0.334
FPG, mg/dL	106 [92, 129]	114 [99, 148]	93 [89, 106]	< 0.001
HbA1c, %	6.3 [5.8, 7.0]	6.8 [6.1, 7.3]	5.9 [5.6, 6.1]	< 0.001
BUN, mg/dL	16.9 [13.8, 20.8]	17.4 [13.9, 21.0]	16.1 [13.4, 20.8]	0.354
Cr, mg/dL	0.88 [0.76, 1.07]	0.87 [0.76, 1.50]	0.90 [0.76, 1.12]	0.532
CysC, mg/L	1.07 [0.93, 1.32]	1.05 [0.93, 1.30]	1.14 [0.90, 1.36]	0.529
LVEF, %	61.9 [52.0, 69.3]	62.2 [50.7, 69.8]	61.3 [53.6, 68.4]	0.700

Values are mean ± standard deviation (SD). *WRF* worsening renal function, *ACEI* angiotensin-converting enzyme inhibitor, *ARB* angiotensin II receptor blocker, *Hb* hemoglobin, *hs-CRP* high-sensitivity C-reactive protein, *LDL-C* low-density lipoprotein cholesterol, *HDL-C* high-density lipoprotein cholesterol, *TG* triglycerides, *UA* uric acid, *FPG* fasting plasma glucose, *HbA1c* glycated hemoglobin, *BUN* blood urea nitrogen, *Cr* creatinine, *eGFR* estimated glomerular filtration rate, *LVEF* left ventricular ejection fraction, *DM* diabetes mellitus, *CysC* cystatin C

ACEI and ARB were prescribed in 56 (29%) and 82 (42%) patients in the DM group and 20 (20%) and 52 (51%) in the non-DM group, respectively. However, no patients were taking both ACEI and ARB. Additionally, no significant differences in baseline eGFR, Cr, and CysC levels were found between the DM and non-DM groups, and the mean volume of contrast media did not differ between groups.

### Association between DM and change in renal function

**Fig. 1:**
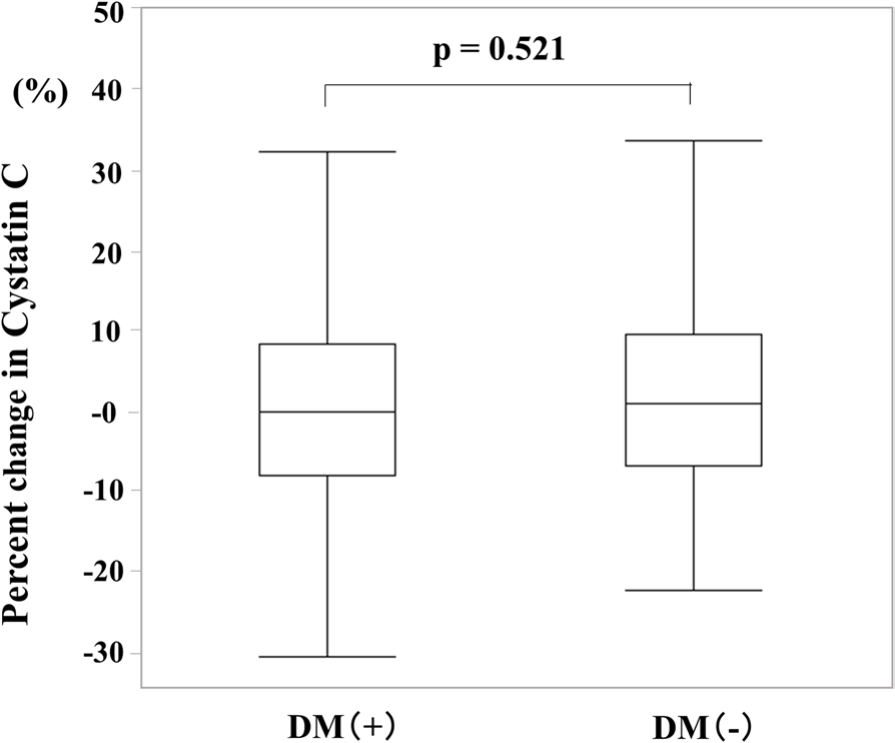
Comparison of the changes in cystatin C among patients with and without DM

We compared the %CysC between groups, and no significant differences in the %CysC were found between the DM and non-DM groups (median: 0%, IQR: -7.9% to 8.5% vs. median: 1.1%, IQR: -6.6% to 9.6%, *p* = 0.521) (Fig. 1). The patients with WRF after PCI were 100 (51%) and 59 (58%) in the DM and non-DM groups, respectively (*p* = 0.261).

### Influence of baseline characteristics on WRF after PCI in the DM and non-DM groups

Tables 2 and 3 present the results of univariate logistic analysis for WRF following PCI in the DM and non-DM groups. In the DM group, age (OR: 1.04, 95% CI: 1.01–1.08, *p* = 0.020) and the use of β-blocker (OR: 2.04, 95% CI: 1.16–3.63, *p* = 0.015) positively correlated with WRF. Body mass index (BMI) (OR: 0.89, 95% CI: 0.81–0.98, *p* = 0.019) and LDL-C (OR: 0.98, 95% CI: 0.97–0.99, *p* = 0.001) were negatively correlated with WRF (Table 2). However, BMI (OR: 1.16, 95% CI: 1.03–1.32, *p* = 0.016), systolic blood pressure (OR: 1.02, 95% CI: 1.01–1.06, *p* = 0.047), calcium channel blocker (OR: 3.36, 95% CI: 1.48–7.89, *p* = 0.004), and ARB (OR: 2.54, 95% CI: 1.14–5.83, *p* = 0.025) positively correlated with WRF in the non-DM group. ACEI negatively correlated with WRF (OR: 0.30, 95% CI: 0.10–0.82, *p* = 0.021) (Table 3). In multivariate logistic analysis for WRF after PCI using relevant factors from univariate analysis including contrast media which was generally considered as a risk factor for renal dysfunction, the use of β-blocker (OR: 2.76, 95% CI: 1.03–7.90, *p* = 0.048) and ACEI (OR: 0.07, 95% CI: 0.01–0.47, *p* = 0.012) were independently correlated with WRF in the DM and non-DM groups, respectively (Tables 2 and 3).

**Table 2:** Logistic regression analyses for WRF in the DM group

	Univariate			Multivariate		
	OR	95% CI	*P*-value	OR	95% CI	*P*-value
Age, y	1.04	1.01–1.08	0.020	1.05	0.98–1.12	0.175
Body mass index	0.89	0.81–0.98	0.019	0.92	0.77–1.08	0.297
Gender: men	0.96	0.51–1.79	0.886			
Contrast media	1.01	0.99–1.02	0.118	1.00	1.00–1.02	0.059
Systolic blood pressure	1.00	0.98–1.02	0.739			
Diastolic blood pressure	1.01	0.98–1.03	0.708			
Dyslipidemia	0.85	0.44–1.62	0.625			
Hyperuricemia	1.21	0.44–3.45	0.717			
Calcium channel blockers	1.01	0.57–1.78	0.967			
ACEI	0.63	0.34–1.18	0.149			
ARB	1.36	0.77–2.41	0.291			
β-blockers	2.04	1.16–3.63	0.015	2.76	1.03–7.90	0.048
Spironolactone	1.40	0.59–3.41	0.446			
Thiazide	1.67	0.38–8.31	0.493			
Loop diuretics	0.89	0.45–1.78	0.747			
Diuretics	1.10	0.58–2.08	0.758			
Hb	0.85	0.71–1.01	0.059			
hs-CRP	1.06	0.93–1.29	0.424			
LDL-C	0.98	0.97–0.99	0.001	0.99	0.97–1.01	0.332
HDL-C	0.99	0.97–1.01	0.379			
TG	1.00	0.99–1.00	0.083			
UA	1.01	0.83–1.24	0.897			
FPG	1.00	0.99–1.01	0.448			
HbA1c	1.12	0.85–1.51	0.429			
BUN	1.01	0.97–1.06	0.608			
Cr	1.26	0.58–2.86	0.558			
eGFR	0.98	0.97–1.00	0.077			
LVEF	0.98	0.96–1.00	0.063			

*DM* diabetes mellitus, *WRF* worsening renal function, *ACEI* angiotensin-converting enzyme inhibitor, *ARB* angiotensin II receptor blocker, *Hb* hemoglobin, *hs-CRP* high-sensitivity C-reactive protein, *LDL-C* low-density lipoprotein cholesterol, *HDL-C* high-density lipoprotein cholesterol, *TG* triglycerides, *UA* uric acid, *FPG* fasting plasma glucose, *HbA1c* glycated hemoglobin, *BUN* blood urea nitrogen, *Cr* creatinine, *eGFR* estimated glomerular filtration rate, *LVEF* left ventricular ejection fraction, *CI* confidence interval, *OR* odds ratio

**Table 3:** Logistic regression analyses for WRF in non-DM group

	Univariate			Multivariate		
	OR	95% CI	*P*-value	OR	95% CI	*P*-value
Age, y	0.99	0.95–1.02	0.631			
Body mass index	1.16	1.03–1.32	0.016	1.03	0.85–1.28	0.747
Gender: men	1.97	0.82–4.81	0.133			
Contrast media	1.00	0.99–1.02	0.457	1.00	0.99–1.02	0.615
Systolic blood pressure	1.02	1.01–1.06	0.047	1.00	0.96–1.04	0.861
Diastolic blood pressure	1.01	0.98–1.05	0.419			
Dyslipidemia	1.75	0.68–4.51	0.244			
Hyperuricemia	0.97	0.37–2.47	0.956			
Calcium channel blockers	3.36	1.48–7.89	0.004	1.07	0.26–4.74	0.923
ACEI	0.30	0.10–0.82	0.021	0.07	0.01–0.47	0.012
ARB	2.54	1.14–5.83	0.025	0.31	0.04–1.48	0.181
β-blockers	1.31	0.59–2.90	0.512			
Spironolactone	0.36	0.09–1.29	0.127			
Thiazide	0.70	0.08–6.02	0.725			
Loop diuretics	0.78	0.26–2.42	0.666			
Diuretics	0.73	0.28–1.95	0.592			
Hb	1.12	0.93–1.37	0.238			
hs-CRP	0.63	0.24–0.97	0.156			
LDL-C	0.99	0.98–1.01	0.875			
HDL-C	1.01	0.98–1.04	0.672			
TG	1.00	0.99–1.01	0.510			
UA	1.01	0.78–1.30	0.939			
FPG	1.01	0.98–1.04	0.529			
HbA1c	1.44	0.56–3.92	0.456			
BUN	0.95	0.88–1.02	0.132			
Cr	0.66	0.19–1.89	0.444			
eGFR	1.02	0.99–1.04	0.157			
LVEF	1.02	0.99–1.06	0.232			

*DM* diabetes mellitus, *WRF* worsening renal function, *ACEI* angiotensin-converting enzyme inhibitor, *ARB* angiotensin II receptor blocker, *Hb* hemoglobin, *hs-CRP* high-sensitivity C-reactive protein, *LDL-C* low-density lipoprotein cholesterol, *HDL-C* high-density lipoprotein cholesterol, *TG* triglycerides, *UA* uric acid, *FPG* fasting plasma glucose, *HbA1c* glycated hemoglobin, *BUN* blood urea nitrogen, *Cr* creatinine, *eGFR* estimated glomerular filtration rate, *LVEF* left ventricular ejection fraction, *CI* confidence interval, *OR* odds ratio

## Discussion

This study demonstrated that the use of β-blockers positively correlated with WRF in the DM group, whereas ACEI negatively correlated with WRF in the non-DM group. This study underscored the different effects of pre-medications on the WRF after PCI between hypertensive patients with and without DM who received treatment with statins.

Previous studies reported that WRF after PCI, including CIN, is a powerful predictor of cardiovascular events and mortality [2, 3, 13–15]. Therefore, preventing WRF after PCI is important to improve prognosis. Renin–angiotensin–aldosterone (RAS) inhibitors are believed to have a renoprotective effect [16]. Additionally, in the NAPLES II trial, CIN prevalence, defined as a > 10% increase in serum CysC, was lower in the atorvastatin group than in the control group [17]. Therefore, statins might also prevent renal dysfunction after using contrast media. DM is well-known as a significant risk factor for CAD and increases overall morbidity and mortality [18]. In patients with DM, renal function is also severely affected due to arteriosclerosis and diabetic nephropathy, and diabetic kidneys are characterized by severe interstitial inflammation [19]. Moreover, DM is believed to be a high-risk factor for developing CIN [20], and several studies have reported that the persistence of renal dysfunction with CIN results in poor prognosis [21]. Therefore, we focused on the impact of medications, including RAS inhibitors, on renal functions in hypertensive patients with DM taking statins.

We previously reported that ACEI had a renoprotective effect in the late phase after PCI rather than ARB [7]. In our report, we considered nitric oxide (NO) a key factor in protecting renal function. ACEI inhibits angiotensin II formation and bradykinin potentiation and increases NO. Interestingly, NO is involved in vascular endothelial function and might be considered to have a renoprotective effect. Furthermore, statins have also been reported to upregulate NO synthase [22, 23] and reduce oxidative stress, which may reduce the progression of renal dysfunction. In this study, we demonstrated that ACEI exhibited a renoprotective effect in the late phase after PCI in patients without DM rather than in those with DM. It was considered that patients with DM had a stronger disorder of NO production than those without DM. Atherosclerosis is characterized by an early reduction in NO [24], and coronary risk factors such as hyperlipidemia and DM are known to impair NO function, which becomes severe with the increase in the number of risk factors. Particularly, DM, characterized by insulin resistance, is associated with an acceleration of atherosclerotic vascular disease and poor outcomes following vascular interventions and is believed to be caused by increasing inflammation and decreasing NO bioavailability [25, 26]. Meininger et al. reported that high glucose levels increase NO synthase (NOS)-dependent superoxide production in human endothelial cells and mediate endothelial NOS dysfunction in endothelial cells [27]. Furthermore, glycated hemoglobin (HbA1c) which showed the long-term glycemic index was positively associated with the severity of CAD even in non-diabetic individuals [28]. We speculated that patients with DM may have severer endothelial dysfunction than those without DM, which may have caused the different effects of ACEI on renal function between patients with and without DM in this study.

Hypertension is an important risk factor for cardiovascular events regardless of its type or presence of left ventricular hypertrophy [29–31]. Furthermore, blood pressure control is associated with several parameters as known to risk factors of coronary artery disease and other cardiac diseases [32, 33]. Therefore, blood pressure control through medication has important implications for the prognosis of heart disease. β-blockers have an important dual role, such as antihypertensive effect and providing optimal cardiovascular protection in patients with hypertension. They are also important drugs for optimal medical therapy for ischemic heart disease. However, to our knowledge, only a few published reports of β-blockers-associated kidney injury after PCI. In our study, we found that β-blockers were the independent risk factor for WRF in the late phase after PCI in hypertensive patients with DM taking statins. There might be a few potential mechanisms in this association between β-blockers and WRF. The effect of the lowering heart rate by β-blockers will play an important role for heart failure. However, the decrease in heart rate may lead to relatively higher central aortic systolic pressure which induce the negative effect on renal function [34]. In addition, animal and cell biology studies showed that β-blockers could have profibrotic effects due to activate the production of TGF-β and collagen I and III [35]. Furthermore, β-blockers reportedly worsen insulin sensitivity, alter lipid metabolism, and cause weight gain [36]. Disorders of insulin sensitivity and lipid metabolism have also been reported to result in endothelial dysfunction. Owing to those mechanisms, using β-blockers might worsen renal function in patients with DM. We summarized the results of this study and previous reports which we have referenced in this study in Table 4.

**Table 4:** Summary of this study and previous reports for renal function and endothelial function

	Protective	Worsening
Renal function	RAS inhibitor [6, 14] Statin [7, 8, 15] Nitric oxide ACEI (non-DM patients) [This study]	DM [18] Contrast media Hypertension β-blockers (DM patients) [This study]
Endothelial function	Nitric oxide	DM Insulin resistance Lipid metabolism

*DM* diabetes mellitus, *ACEI* angiotensin-converting enzyme inhibitor, *RAS* Renin–angiotensin–aldosterone

Currently, there is insufficient evidence of the benefit of β-blockers use for stable angina pectoris patients without left ventricular dysfunction. In addition, β-blockers are not first-line agents for hypertensive patients. Based on the results of the present study, we believe that careful consideration should be needed to use β-blockers in DM patients complicated stable angina pectoris who require PCI, except in patients with left ventricular dysfunction who could clearly benefit from use of β-blockers.

This study had some limitations. First, all subjects underwent elective PCI and follow-up coronary angiography 9 months after PCI at our hospital and this study was a retrospective study with a relatively small number of patients. Therefore, a large-scale prospective study should assess the effect of ACEI and β-blockers on renal function over a longer time after PCI. Second, only patients with mild to moderate renal dysfunction were recruited in this study. Therefore, the impact of ACEI and β-blockers on renal function in the late phase after PCI in patients with severe renal dysfunction should be investigated. Third, all medications that the patients took were checked at the time of admission. However, we could not completely confirm that the patients took all medicines regularly during study period.

## Conclusions

The use of β-blockers was the independent risk factor for WRF in hypertensive patients with DM in the late phase after PCI for stable angina pectoris who underwent statin therapy, whereas ACEI had a renoprotective effect in patients without DM.

## Data Availability

The datasets used and/or analyzed during the current study are available from the corresponding author upon reasonable request.

## Abbreviations

ACEI: Angiotensin-converting enzyme inhibitor
DM: Diabetes mellitus
CAD: Coronary artery disease
PCI: Percutaneous intervention
CysC: Cystatin
%CysC: Percent change in cCysC
OR: Odds ratio
CI: Confidence interval
IQR: Interquartile range
WRF: Worsening renal function
AKI: Acute kidney injury
CKD: Chronic kidney disease
ARB: Angiotensin II receptor blockers
CIN: Contrast-induced nephropathy
LDL-C: Low-density lipoprotein cholesterol
Cr: Creatinine
eGFR: Estimated glomerular filtration rate
HDL-C: High-density lipoprotein cholesterol
BMI: Body mass index
RAS: Renin–angiotensin–aldosterone
NO: Nitric oxide
NOS: NO synthase

## References

[1] Jiménez-Navarro MF, López-Jiménez F, Barsness G, Lennon RJ, Sandhu GS, Prasad A (2015). Long-term prognosis of complete percutaneous coronary revascularization in patients with diabetes with multivessel disease. Heart.

[2] McCullough PA, Wolyn R, Rocher LL, Levin RN, O'Neill WW (1997). Acute renal failure after coronary intervention: incidence, risk factors, and relationship to mortality. Am J Med.

[3] Rihal CS, Textor SC, Grill DE, Berger PB, Ting HH, Best PJ (2002). Incidence and prognostic importance of acute renal failure after percutaneous coronary intervention. Circulation.

[4] Murata N, Kaneko H, Yajima J, Oikawa Y, Oshima T, Tanaka S (2015). The prognostic impact of worsening renal function in Japanese patients undergoing percutaneous coronary intervention with acute coronary syndrome. J Cardiol.

[5] Go AS, Chertow GM, Fan D, McCulloch CE, Hsu CY (2004). Chronic kidney disease and the risks of death, cardiovascular events, and hospitalization. N Engl J Med.

[6] Aktan A, Güzel T (2023). Prognostic value of age, creatinine, and left ventricular ejection fraction risk score in patients evaluated with fractional flow reserve: a cross-sectional study. Rev Assoc Med Bras (1992)..

[7] Kanda D, Takumi T, Miyata M, Tokushige A, Sonoda T, Yoshino S (2016). Angiotensin-converting enzyme inhibitor prevents the worsening of renal function in the late phase after percutaneous coronary intervention. J Atheroscler Thromb.

[8] Sandhu S, Wiebe N, Fried LF, Tonelli M (2006). Statins for improving renal outcomes: a meta-analysis. J Am Soc Nephrol.

[9] Barbieri L, Verdoia M, Schaffer A, Nardin M, Marino P, De Luca G (2014). The role of statins in the prevention of contrast induced nephropathy: a meta-analysis of 8 randomized trials. J Thromb Thrombolysis.

[10] Matsuo S, Imai E, Horio M, Yasuda Y, Tomita K, Nitta K (2009). Revised equations for estimated GFR from serum creatinine in Japan. Am J kidney Dis.

[11] KDIGO (2012). clinical practice guideline for the evaluation and management of chronic kidney disease. Kidney Int Suppl.

[12] The International Expert Committee (2009). International Expert Committee Report on the role of the A1c assay in the diagnosis of diabetes. Diabetes Care.

[13] Best PJ, Lennon R, Ting HH, Bell MR, Rihal CS, Holmes DR (2002). The impact of renal insufficiency on clinical outcomes in patients undergoing percutaneous coronary interventions. J Am Coll Cardiol.

[14] Gupta R, Gurm HS, Bhatt DL, Chew DP, Ellis SG (2005). Renal failure after percutaneous coronary intervention is associated with high mortality. Catheter Cardiovasc Interv.

[15] Güzel T, Aktan A, Demir M, Özbek M, Aslan B (1992). Relationship between contrast-induced nephropathy and long-term mortality after percutaneous coronary intervention in patients with chronic coronary total occlusion. Rev Assoc Med Bras.

[16] Zandi-Nejad K, Brenner BM (2005). Primary and secondary prevention of chronic kidney disease. J Hypertens.

[17] Quintavalle C, Fiore D, De Micco F, Visconti G, Focaccio A, Golia B (2012). Impact of a high loading dose of atorvastatin on contrast-induced acute kidney injury. Circulation.

[18] Bertoluci MC, Rocha VZ (2017). Cardiovascular risk assessment in patients with diabetes. Diabetol Metab Syndr.

[19] Najafian B, Alpers CE, Fogo AB (2011). Pathology of human diabetic nephropathy. Contrib Nephrol.

[20] Toprak O, Cirit M, Yesil M, Bayata S, Tanrisev M, Varol U (2007). Impact of diabetic and pre-diabetic state on development of contrast-induced nephropathy in patients with chronic kidney disease. Nephrol Dial Transplant.

[21] McCullough PA, Adam A, Becker CR, Davidson C, Lameire N, Stacul F (2006). Epidemiology and prognostic implications of contrast-induced nephropathy. Am J Cardiol.

[22] Athyros VG, Kakafika AI, Tziomalos K, Karagiannis A, Mikhailidis DP (2009). Pleiotropic effects of statins–clinical evidence. Curr Pharm Des.

[23] O'Driscoll G, Green D, Taylor RR (1997). Simvastatin, an HMG-coenzyme A reductase inhibitor, improves endothelial function within 1 month. Circulation.

[24] Chen JY, Ye ZX, Wang XF, Chang J, Yang MW, Zhong HH (2017). Nitric oxide bioavailability dysfunction involves in atherosclerosis. Biomed Pharmacother.

[25] Barbato JE, Zuckerbraun BS, Overhaus M, Raman KG, Tzeng E (2005). Nitric oxide modulates vascular inflammation and intimal hyperplasia in insulin resistance and the metabolic syndrome. Am J Physiol Heart Circ Physiol.

[26] Karakayali M, Omar T, Artac I, Rencuzogullari İ, Karabag Y, Demir O (2023). The relationship between the systemic immune-inflammation index and reverse-dipper circadian pattern in newly diagnosed hypertensive patients. J Clin Hypertens (Greenwich).

[27] Meininger CJ, Marinos RS, Hatakeyama K, Martinez-Zaguilan R, Rojas JD, Kelly KA (2000). Impaired nitric oxide production in coronary endothelial cells of the spontaneously diabetic BB rat is due to tetrahydrobiopterin deficiency. Biochem J.

[28] Kis M, Guzel T (2022). Relationship between hemoglobin A1c and fractional flow reserve lesion severity in non-diabetic patients. J Coll Physicians Surg Pak.

[29] Karakayali M, Artac I, Omar T, Rencuzogullari İ, Karabag Y, Cinar T (2023). The association between frontal QRS-T angle and reverse dipper status in newly diagnosed hypertensive patients. Blood Press Monit.

[30] Altunova M, Püşüroğlu H, Karakayalı M, Şahin AA, Demir AR, Yılmaz E (2022). Relationship between fragmented QRS complex and long-term cardiovascular outcome in patients with essential hypertension. Anatol J Cardiol.

[31] Kis M, Dogan Y, Yildirim A, Güzel T, Bekar L, Akhan O (2022). Evaluation of demographic, clinical, and aetiological data of patients admitted to cardiology clinics and diagnosed with left ventricular hypertrophy in Turkish population (LVH-TR). Acta Cardiol..

[32] Aktas G, Khalid A, Kurtkulagi O, Duman TT, Bilgin S, Kahveci G (2022). Poorly controlled hypertension is associated with elevated serum uric acid to HDL-cholesterol ratio: a cross-sectional cohort study. Postgrad Med.

[33] Mansiroglu AK, Coşgun M, Sincer I, Gunes Y (2021). The role of baseline and post-treatment frontal QRS-T angle for detecting arterial blood pressure control. Clin Exp Hypertens.

[34] Dahlöf B, Devereux RB, Kjeldsen SE, Julius S, Beevers G, de Faire U, LIFE Study Group (2002). Cardiovascular morbidity and mortality in the losartan intervention for endpoint reduction in hypertension study (LIFE): a randomised trial against atenolol. Lancet..

[35] Akutsu S, Shimada A, Yamane A (2006). Transforming growth factor betas are upregulated in the rat masseter muscle hypertrophied by clenbuterol, a beta2 adrenergic agonist. Br J Pharmacol.

[36] Manrique C, Johnson M, Sowers JR (2010). Thiazide diuretics alone or with beta-blockers impair glucose metabolism in hypertensive patients with abdominal obesity. Hypertension.

